# Elevation of serum sphingosine-1-phosphate attenuates impaired cardiac function in experimental sepsis

**DOI:** 10.1038/srep27594

**Published:** 2016-06-09

**Authors:** Sina M. Coldewey, Elisa Benetti, Massimo Collino, Josef Pfeilschifter, Christoph Sponholz, Michael Bauer, Andrea Huwiler, Christoph Thiemermann

**Affiliations:** 1 Department of Anesthesiology and Intensive Care Medicine, University Hospital Jena, Jena, Germany; 2 Center for Sepsis Control and Care, University Hospital Jena, Jena, Germany; 3 Department of Drug Science & Technology, University of Turin, Turin, Italy; 4 Pharmazentrum Frankfurt/ZAFES, University Hospital Frankfurt, Frankfurt am Main, Germany; 5 Institute of Pharmacology, University of Bern, Bern, Switzerland; 6 The William Harvey Research Institute, Barts & The London School of Medicine & Dentistry, Queen Mary University of London, London, UK

## Abstract

Serum levels of the lipid mediator sphingosine-1-phosphate (S1P) are reduced in septic patients and are inversely associated with disease severity. We show that serum S1P is reduced in human sepsis and in murine models of sepsis. We then investigated whether pharmacological or genetic approaches that alter serum S1P may attenuate cardiac dysfunction and whether S1P signaling might serve as a novel theragnostic tool in sepsis. Mice were challenged with lipopolysaccharide and peptidoglycan (LPS/PepG). LPS/PepG resulted in an impaired systolic contractility and reduced serum S1P. Administration of the immunomodulator FTY720 increased serum S1P, improved impaired systolic contractility and activated the phosphoinositide 3-kinase (PI3K)-pathway in the heart. Cardioprotective effects of FTY720 were abolished following administration of a S1P receptor 2 (S1P_2_) antagonist or a PI3K inhibitor. Sphingosine kinase-2 deficient mice had higher endogenous S1P levels and the LPS/PepG-induced impaired systolic contractility was attenuated in comparison with wild-type mice. Cardioprotective effects of FTY720 were confirmed in polymicrobial sepsis. We show here for the first time that the impaired left ventricular systolic contractility in experimental sepsis is attenuated by FTY720. Mechanistically, our results indicate that activation of S1P_2_ by increased serum S1P and the subsequent activation of the PI3K-Akt survival pathway significantly contributes to the observed cardioprotective effect of FTY720.

In sepsis life-threatening organ dysfunction occurs due to a dysregulated host response to infection^1^. Among the various organ systems that fail in sepsis, the cardiovascular system plays a prominent role. The development of left ventricular systolic dysfunction is common in patients with severe sepsis and is associated with increased mortality^2^,^3^. However, the pathophysiology of septic cardiomyopathy is not well understood^4^, but may be a consequence of dysregulated systemic inflammation.

Bioactive lipids are increasingly recognized as key mediators determining progression and resolution of inflammation^5^,^6^. One molecule that has been attributed a key role in inflammation is the lipid sphingosine-1-phosphate (S1P). This biologically active phospholipid mediator acts as a ligand of five different high affinity S1P receptors, denoted S1P_1-5_^7^, which belong to the superfamily of G protein-coupled receptors (GPCR) and couple to a variety of signal transduction cascades either through G_i_, G_q_, G_12/13_ or small G proteins^8^. This diversity of S1P receptor-triggered signal transduction implicates a multitude of physiological and pathophysiological functions of extracellular S1P including the promotion of cell growth and survival, cell migration, but also the enhancement of endothelial barrier function^9^,^10^,^11^,^12^. Consequently, targeting of S1P signaling by the development of specific pharmacological tools may have therapeutic potential in diseases associated with dysfunction of endothelial barrier^13^,^14^,^15^,^16^, such as sepsis.

Most notably, it has been reported recently that S1P serum levels are decreased in patients with sepsis and are inversely associated with disease severity^17^. However, it remains unclear if this observation is associative or whether there is a true cause-effect relationship between S1P levels and cardiac (dys)function. Thus, S1P (and/or its signaling pathway) may serve as a biomarker or even as a theragnostic target in patients with sepsis.

We show that serum S1P is reduced in human and experimental murine sepsis. We then aimed to elucidate the role of S1P and its therapeutic potential in ameliorating sepsis-induced cardiomyopathy in a reverse-translational approach. To address our objectives, we employed two different murine models of experimental sepsis complementing each other by recapitulating some individual features of the human disease. Mice received either cell wall fragments from Gram-negative (lipopolysaccharide, LPS) and Gram-positive (peptidoglycan, PepG) bacteria to induce severe multiple organ dysfunction (MOD) including impaired systolic contractility of the heart (for mechanistic studies) or they underwent cecal-ligation and puncture (CLP) to induce septic cardiomyopathy (for proof of principle studies). Specifically, we investigated the effects and underlying mechanisms of pharmacological (administration of the immunomodulator FTY720) or genetic (sphingosine kinase-2 deficient (SPHK-2^−/−^) mice) approaches to alter S1P receptor signaling on the impaired systolic contractility and S1P levels in experimental sepsis.

## Methods

Additional details relating to materials and methodology are provided in the online data supplement.

### Use of human subjects–ethic statement

The methods using human subjects were carried out in accordance with the approved guidelines. Specifically, following approval of the local ethics committee of Jena University Hospital, Germany (application no 2160-11/07, 2712-12/09), written informed consent for blood sampling, sample analysis and data collection was obtained from all patients or legal surrogates before enrolment.

### Human pilot study

Human samples were collected from a large cohort of septic patients admitted to the multidisciplinary intensive care unit of Jena University Hospital^18^. Samples were snap frozen and stored at −80 °C in the certified biobank of the Center for Sepsis Control & Care at Jena University Hospital. All samples were prospectively collected within 24 h or 72 h (±6 h) after the patients fulfilled criteria of severe sepsis/septic shock according to ACCP/SCCM matching the newly introduced criteria for sepsis/septic shock (in accordance to the sepsis-3)^1^. To enable best possible homogeneity, septic patients (n = 19) were selected based on fulfilling anastomosis insufficiency after major abdominal surgery as a well-defined focus of this study. We included 28-d survivors (n = 9) and non-survivors (n = 10). Further information on patients characteristics are displayed in Supplementary Table S1.

Control patients (n = 11) underwent minimal invasive direct coronary bypass surgery (n = 5) or cardiac surgery with cardio-pulmonary bypass (n = 6). Serum samples of each patient were collected prior to operation (after cannulation) and early postoperative (suture), reflecting an age-matched cohort with signs of systemic inflammation.

### Use of experimental animals–ethic statement

The experimental protocols using animals were carried out in accordance with the approved guidelines and were approved by the Home Office, London, UK (project licenses reference numbers: PPL 70/6525, PPL 70/7348; personal license number: PIL 70/22807). The local ‘Animal Use and Care Committee’ approved animal experiments in accordance with the derivatives of both, the ‘Home Office guidance on the Operation of Animals (Scientific Procedures) Act 1986’, and the ‘Guide for the Care and Use of Laboratory Animals’ of the National Research Council.

### Model of multiple organ dysfunction caused by LPS/PepG co-administration

This study was carried out on 2-month-old male C57BL/6J mice (Charles River, Kent, UK) or sphingosine kinase-2 deficient (SPHK-2^−/−^, Genoway, Lyon, France) mice weighing 25–30 g, receiving a standard diet and water *ad libitum*. Infomation on generation of SPHK-2^−/−^ mice can be found in the Supplementary. C57BL/6J wild-type or SPHK-2^−/−^ mice received *i.p.*-injections of LPS (9 mg/kg)/PepG (1 mg/kg) or its vehicle (0.9% saline). Sham mice were not subjected to LPS/PepG, but were otherwise treated in the same way. At 1 h after LPS/PepG challenge, mice were treated with FTY720 (0.1 mg/kg *i.v.*) or its vehicle (10% DMSO). To elucidate the role of different S1P receptors in the observed effects of FTY720, mice received (45 min after LPS/PepG and 15 min prior to FTY720) the selective phosphatidylinositol 3 (PI3)-kinase inhibitor LY294002 (0.3 mg/kg *i.v.*) or the selective S1P_2_ receptor antagonist JTE 013 (1 mg/kg *i.v.*) or (1 h after LPS/PepG) the selective S1P_1_ receptor agonist SEW2871 (1 mg/kg *i.v.*) or vehicle (10% DMSO).

### Model of polymicrobial sepsis caused by cecal ligation and puncture (CLP)

This study was carried out on 8-month-old male C57BL/6J mice (Charles River, Kent, UK) weighing 35–45 g, receiving a standard diet and water *ad libitum*. C57BL/6J mice underwent CLP (18-G needle, double puncture) or sham operation. Ringer´s solution (1 ml/mouse *s.c.*) was administered after surgery. Mice received antibiotic (imipenem/cilastin; 20 mg/kg *i.p.*) and analgesic (buprenorphine; 0.05 mg/kg *s.c.*) therapy as well as fluid resuscitation (Ringer´s solution; 0.5 ml/mouse *s.c.*) 6 and 18 h after surgery^19^,^20^. Sham mice were not subjected to CLP, but were otherwise treated the same way. At 1 h after CLP, mice were treated with FTY720 (0.1 mg/kg *i.v.*) or vehicle (10% DMSO).

### Quantification of organ dysfunction/injury

Cardiac function was assessed in mice subjected to LPS/PepG or CLP at 18 h or 24 h, respectively, *via* echocardiography using a Vevo-770 imaging system (Visual Sonics, Toronto, Canada)^20^,^21^. Then, the experiment was terminated and organ and blood samples were collected for quantification of organ dysfunction and injury.

### Quantification of S1P

Lipid extraction in serum samples was performed according to Bligh and Dyer^22^. S1P concentration was determined by liquid chromatography-coupled tandem mass spectrometry (LC/MS/MS) in a blinded fashion^23^.

### Immunoblot analysis

Semi-quantitative immunoblot analyses were carried out in mouse heart tissues as described previously^20^.

### Statistics

Values are presented as box and whisker (min to max) with mean or as mean ± standard error of the mean (SEM) of *n* observations, where *n* represents the number of patients/animals studied. Due to the relatively low n-numbers, data (assumed to be not normally distributed) was assessed by Kruskal-Wallis test and Dunn´s test (corrected for multiple comparisons) unless otherwise stated. A *P*-value of less than 0.05 was considered to be statistically significant.

## Results

Additional results are provided in the online *Supplementary*.

### S1P serum concentrations are decreased in patients with sepsis and in mice challenged with LPS/PepG.

**Figure 1:**
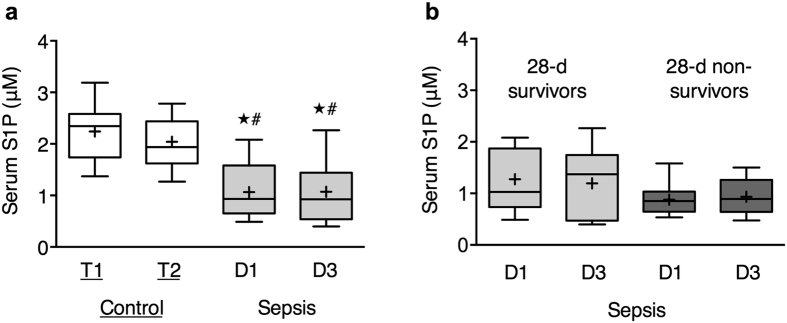
Decreased S1P serum concentrations in patients with sepsis. **(a,b)** S1P serum levels were assessed by LC/MS/MS in control patients (undergoing cardiac surgery prior to operation (T1 n = 11) and early postoperative (T2 n = 11) and in patients with severe sepsis/septic shock at day 1 (D1 n = 19) and day 3 (D3 n = 17) after diagnosis. (**b)** Septic patients were divided in 28-d survivors (D1 n = 9; D3 n = 9) and 28-d non-survivors (D1 n = 10; D3 n = 8). Data are expressed as box and whisker min to max for n number of observations. + = mean value. *P < 0.05 sepsis vs. control T1 ^#^P < 0.05 sepsis vs. control T2 (Kruskall-Wallis test with Dunn´s multiple comparisons test).

S1P serum levels are significantly lower in septic patients compared to control patients (*P* = 0.0006, control T1 vs. sepsis D1). This effect was independent of the chosen time point (day 1 or day 3 after sepsis diagnosis) of blood sampling (Fig. 1a). A subanalysis revealed lower S1P levels in 28-day non-survivors compared to survivors, however, this effect was not significant (Fig. 1b).

**Figure 2:**
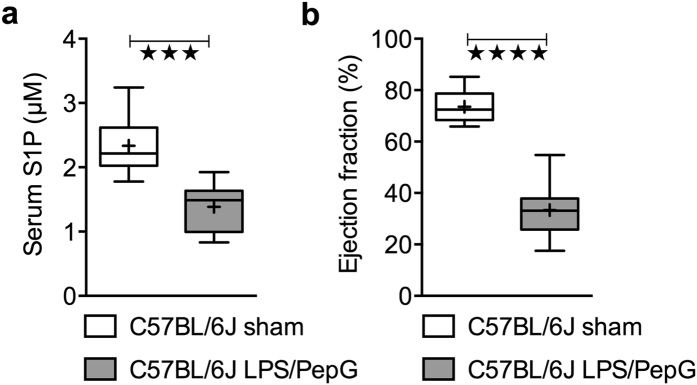
Effect of LPS/PepG on S1P serum levels and on cardiac function in C57BL/6J wild type mice. At 18 h after LPS/PepG or vehicle (sham) administration to 2-month-old male C57BL/6J wild type mice (**a**) S1P serum levels were assessed by LC/MS/MS, or (**b**) the percentage ejection fraction was assessed by echocardiography. The following groups were studied: sham + vehicle (n = 8); LPS/PepG + vehicle (n = 8). Data are expressed as means ± SEM, ***P = 0.005 vs. sham + vehicle, ****P = 0.0001 vs. sham + vehicle (unpaired t-test).

When compared to sham-operated mice, LPS/PepG challenge caused a significant (*P* = 0.0005) fall in serum S1P levels (Fig. 2a) as well as a significant (*P* < 0.0001) decline in percentage ejection fraction (EF) (Fig. 2b) and, hence, cardiac dysfunction.

### Cardiac dysfunction following LPS/PepG co-administration is attenuated by FTY720 treatment

**Figure 3:**
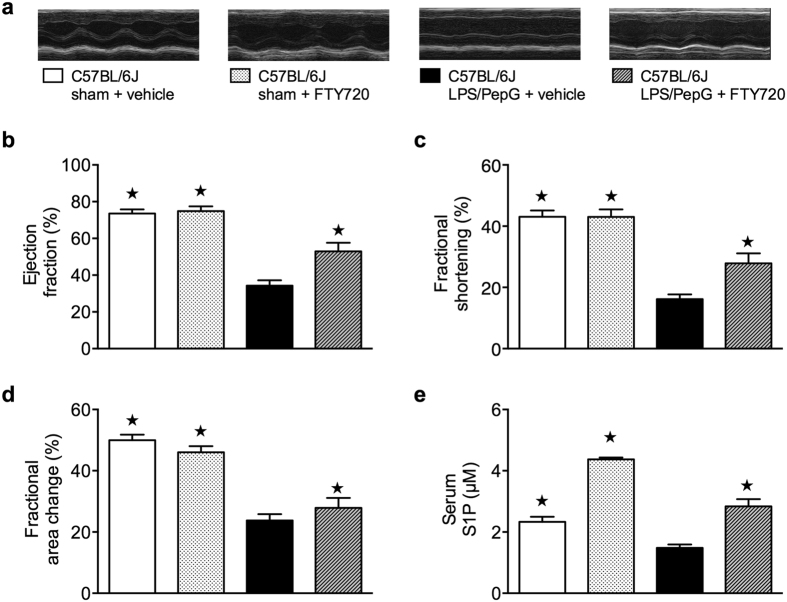
Effect of LPS/PepG co-administration and treatment with FTY720 on cardiac function and S1P serum levels in C57BL/6J wild type mice. (**a**) Representative M-mode echocardiograms and legend of all groups studied. (**b**) Percentage ejection fraction, (**c**) fractional shortening (**d**), fractional area change were assessed via echocardiography and (**e**) S1P serum levels were assessed by LC/MS/MS 18 h subsequent to vehicle administration (sham) or LPS/PepG co-administration in 2-month-old male C57BL/6J wild type mice. At 1 h after LPS/PepG challenge mice were treated either with FTY720 (0.1 mg/kg) or vehicle (10% DMSO). The following groups were studied: C57BL/6J sham + vehicle (n = 8); C57BL/6J sham + FTY720 (n = 3); C57BL/6J LPS/PepG + vehicle (n = 14); C57BL/6J LPS/PepG + FTY720 (n = 15). Data are expressed as means ± SEM for *n* number of observations. *P < 0.05 vs. C57BL/6J LPS/PepG + vehicle (Kruskall-Wallis test with Dunn´s multiple comparisons test).

The immunomodulator FTY720 is a structural analogue of S1P and acts in its phosphorylated isoform as an unselective agonist on S1P_1_ and S1P_3-5_ and a selective functional antagonist on S1P_1_^24^. There is evidence that FTY720, enhances serum S1P levels by inhibiting S1P lyase activity^25^. Thus, we investigated the effects of FTY720 on both serum S1P levels and cardiac function in mice challenged with LPS/PepG. There were no significant differences in percentage EF, fractional shortening (FS) and fractional area change (FAC) in sham animals treated with FTY720^26^ or vehicle (Fig. 3a–d). When compared to the sham animals, mice subjected to LPS/PepG demonstrated a significant reduction of percentage EF (*P* = 0.0001), FS (*P* < 0.0001) and FAC (*P* < 0.0001), indicating impaired systolic contractility *in vivo* (Fig. 3a–d). Delayed intravenous administration of FTY720 (0.1 mg/kg) 1 h after LPS/PepG challenge significantly attenuated this impaired systolic contractility, indicated by significantly higher values for EF (*P* = 0.0332), FS (*P* = 0.0378) and FAC (*P* < 0.0482) (Fig. 3a–d).

### S1P serum level following LPS/PepG co-administration are increased by FTY720 treatment

Administration of FTY720 caused a significant rise in serum S1P levels in both sham-operated animals and animals challenged with LPS/PepG (*P* < 0.0001) (Fig. 3e). These data (Fig. 3) support the view that the increase in serum S1P afforded by FTY720 might contribute to an improvement in cardiac function.

### Effect of LPS/PepG co-administration on cardiac function in SPHK-2 deficient mice

Serum S1P-levels are higher in sphingosine kinase 2 deficient (SPHK-2^−/−^) mice^27^,^28^,^29^. If serum S1P levels do indeed (as we propose) protect the heart against the cardiac dysfunction caused by LPS/PepG, then one would expect SPHK-2^−/−^ mice to be more resistant to the cardiac dysfunction caused by LPS/PepG.

**Figure 4:**
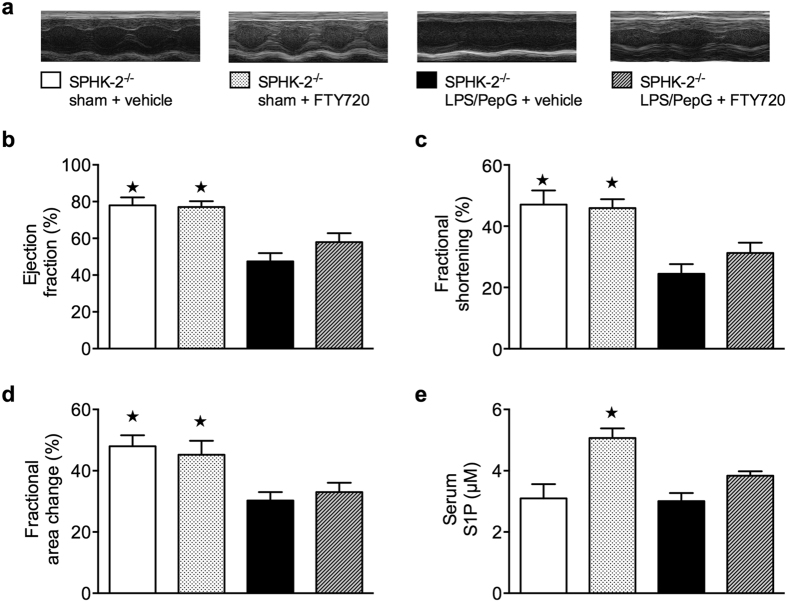
Effect of LPS/PepG co-administration and treatment with FTY720 on cardiac function and S1P serum levels in sphingosine kinase-2 deficient mice. (**a**) Representative M-mode echocardiograms and legend of all groups studied. (**b**) Percentage ejection fraction, (**c**) fractional shortening (**d**), fractional area change were assessed via echocardiography and (**e**) S1P serum levels were assessed by LC/MS/MS 18 h subsequent to vehicle administration (sham) or LPS/PepG co-administration in 2-month-old male SPHK-2^−/−^ mice. At 1 h after LPS/PepG challenge mice were treated either with FTY720 (0.1 mg/kg) or vehicle (10% DMSO). The following groups were studied: SPHK-2^−/−^ sham + vehicle (n = 6); SPHK-2^−/−^ sham + FTY720 (n = 6), SPHK-2^−/−^ LPS/PepG + vehicle (n = 16), SPHK-2^−/−^ LPS/PepG + FTY720 (n = 14). Data are expressed as means ± SEM for *n* number of observations. *P < 0.05 vs. SPHK-2^−/−^ LPS/PepG + vehicle (Kruskall-Wallis test with Dunn´s multiple comparisons test).

When compared to SPHK-2^−/−^ sham mice, SPHK-2^−/−^ mice subjected to LPS/PepG developed a significant reduction of percentage EF (*P* = 0.0059), FS (*P* = 0.0063) and FAC (*P* = 0.0148) and, thus, impaired systolic contractility (Fig. 4a–d). Most notably, systolic contractility was significantly less impaired in SPHK-2^−/−^ mice than in wild-type mice (Figs 3a–d and 4a–d) subjected to LPS/PepG co-administration (Mann Whitney test EF C57BL/6J LPS/PepG vs. Sphk2^−/−^ LPS/PepG, (*P* = 0.0422)), indicating a protective effect mediated by SPHK-2 deficiency. Serum S1P-levels were higher in sham-operated SPHK-2^−/−^ mice (3.099 ± 0.4644) than in sham-operated wild-type mice (2.334 ± 0.1644) (Figs 4e and 3e). While LPS/PepG challenge in wild-type mice (and sepsis in patients) resulted in a significant decline in serum S1P levels, this was not observed in SPHK-2^−/−^ mice challenged with LPS/PepG (*P *> 0.999) (Fig. 4e). This supports the view that higher S1P levels are associated with a better cardiac function. Consequently, delayed intravenous administration of FTY720 (0.1 mg/kg) 1 h after LPS/PepG challenge in SPHK2^−/−^ mice did not significantly attenuate percentage EF, FS and FAC and serum S1P (Fig. 4a–e).

### Effect of LPS/PepG co-administration and treatment with FTY720 on Akt, eNOS and ERK1/2 phosphorylation in murine heart tissue

**Figure 5:**
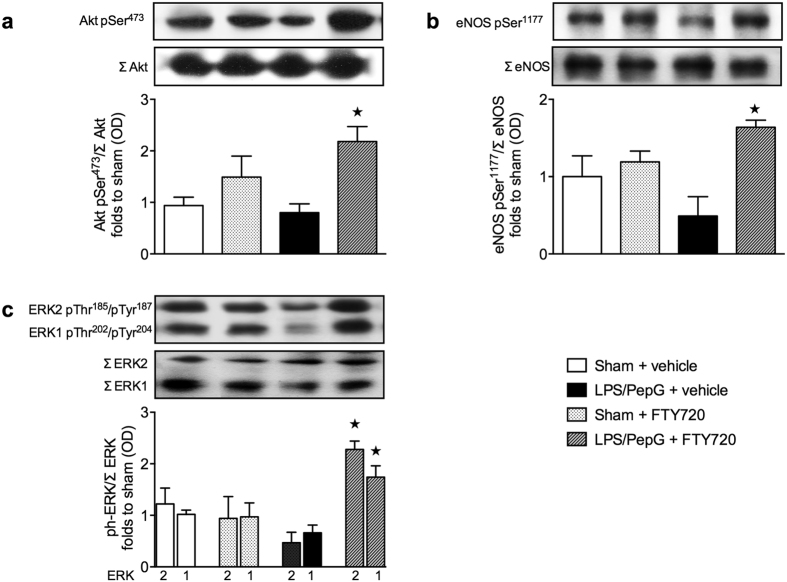
Effect of LPS/PepG co-administration and treatment with FTY720 on Akt, eNOS and ERK1/2 phosphorylation in heart tissue of C57BL/6J wild type mice. At 1 h after administration of LPS/PepG or vehicle (sham), mice were treated with FTY720 (0.1 mg/kg) or vehicle (10% DMSO). Signaling events in heart tissue were assessed at 18 h. Each immunoblot is from a single experiment and is representative of three separate experiments. All values were corrected for the corresponding β-actin band. Densitometric analysis of the bands is expressed as relative optical density (OD) of (**a**) phosphorylated Akt (pSer^473^) corrected for the corresponding total Akt content (Σ Akt) and normalized using the related sham band; (**b**) phosphorylated eNOS (pSer^1177^) corrected for the corresponding total eNOS content (Σ eNOS) and normalized using the related sham band and; (**c**) phosphorylated ERK1 (pThr^202^/pTyr^204^) and ERK2 (pThr^185^/pTyr^187^) corrected for the corresponding total ERK1 or ERK2 content (Σ ERK1 or Σ ERK2) and normalized using the related sham band. Data are expressed as mean ± SEM for *n* number of observations. *P < 0.05 vs. LPS/PepG + vehicle (Kruskall-Wallis test with Dunn´s multiple comparisons test).

Activation of S1P receptors results in activation (phosphorylation) of Akt, eNOS and ERK1/2^30^, while activation of Akt and eNOS improves cardiac function in mice with sepsis^31^ and activation of ERK1/2 mediates cardioprotection^32^. Thus, we investigated the effects of FTY720 on the degree of phosphorylation of Akt on Ser^473^ (Fig. 5a), eNOS on Ser^1177^ (Fig. 5b) and ERK1/2 on Thr^202^/Tyr^204^ and Thr^185^/Tyr^187^ (Fig. 5c), respectively. FTY720 did not affect the degree of phosphorylation of any of the above proteins in sham-operated animals. In contrast, FTY720 significantly increased phosphorylation of Akt on Ser^473^ (*P* = 0.0208) (Fig. 5a), eNOS on Ser^1177^ (*P* = 0.00273) (Fig. 5b) and ERK1/2 on Thr^202^/Tyr^204^ and Thr^185^/Tyr^187^ (*P* = 0.00273) (Fig. 5c) in mice challenged with LPS/PepG.

### Role of S1P_1_ and S1P_2_ on the FTY720 mediated effects on cardiac function in LPS/PepG challenged mice

**Figure 6:**
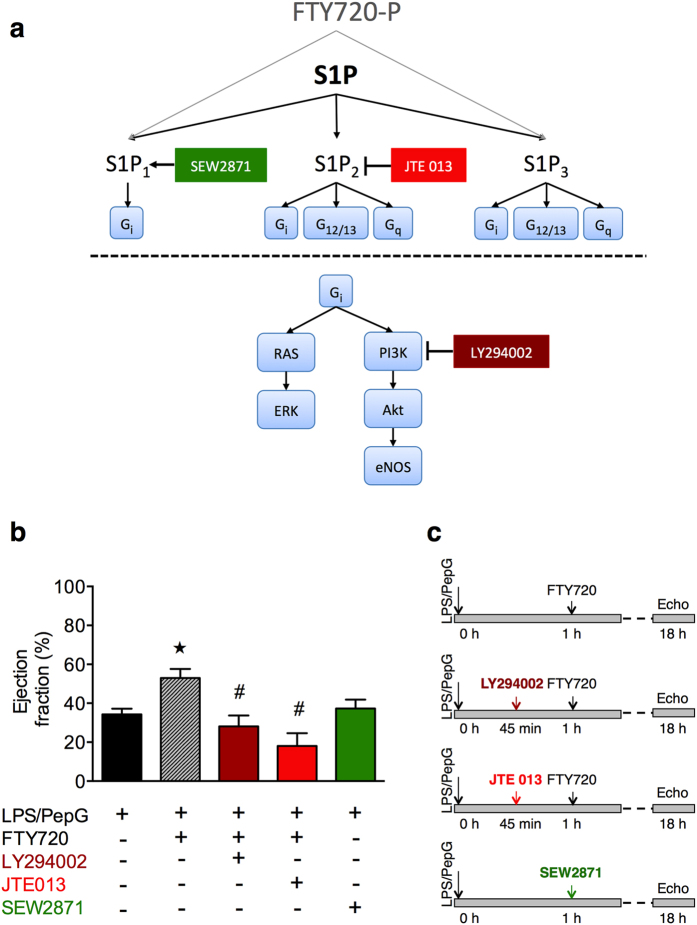
Role of S1P_1_ and S1P_2_ on observed LPS/PepG- and FTY720-mediated effects on cardiac function in mice. (**a**) S1P receptors S1P_1-3_, and the activation of ERK and PI3K/Akt/eNOS through G_i_ coupled signaling pathways. S1P activates S1P receptors. The S1P mimetic FTY720 acts in its phosphorylated isoform as an unselective agonist on S1P_1_ and S1P_3_ and a selective functional antagonist on S1P_1_. S1P_1_ exclusively couples to G_i_, while S1P_2_ and S1P_3_ couple to G_i_, G_12/13_ and G_q_. Coupling of S1P receptors to G_i_ leads to activation of the Ras/ERK pathway and the PI3K/Akt/eNOS pathway. SEW2871: S1P_1_ agonist. JTE 013: S1P_2_ antagonist. LY294002: PI3K inhibitor. S1P: sphingosine-1-phosphate. FTY720-P: phosphorylated FTY720. (**b**) Percentage ejection fraction was assessed via echocardiography 18 h subsequent to LPS/PepG co-administration in 2-month-old male C57BL/6J mice. At 1 h after LPS/PepG challenge mice were treated either with vehicle (10% DMSO) (n = 14), FTY720 (0.1 mg/kg) (n = 15) or the selective S1P_1_ agonist SEW2871 (1 mg/kg *i.v.*) (n = 3). Or mice received (45 min after LPS/PepG and 15 min prior to FTY720) the selective PI3K inhibitor LY294002 (0.3 mg/kg *i.v.*) (n = 6) or the selective S1P_2 _antagonist JTE 013 (1 mg/kg *i.v.*) (n = 6). One animal, which received JTE 013, died and was not included in the statistics. Data are expressed as means ± SEM for *n* number of observations. *P < 0.05 LPS/PepG + FTY720 vs. LPS/PepG + vehicle; ^#^*P* < 0.05 vs. LPS/PepG + FTY720 (Kruskall-Wallis test with Dunn´s multiple comparisons test). **(c)** Summary of the experimental setup for acquisition of data provided in panel **b**.

Activation of the S1P receptors S1P_1_, S1P_2_ or S1P_3_ results in activation of the PI3K/Akt/eNOS pathway (Fig. 6a). The beneficial effects of FTY720 on cardiac dysfunction in LPS/PepG challenged mice was abolished in mice that received either the selective phosphatidylinositol 3 (PI3)-kinase inhibitor LY294002^33^ or the selective S1P_2_ antagonist JTE013^34^ 15 min prior to FTY720 treatment (Fig. 6b,c). Of note, one mouse of the JTE013 treated group died. Administration of the selective S1P_1_ agonist SEW2871^35^ (1 mg/kg *i.v.)* (Fig. 6b,c) or 10 mg/kg (data not shown) at 1 h after LPS/PepG challenge did not significantly attenuate percentage EF, FS and FAC in LPS/PepG challenged mice. These results suggest that activation of S1P_2_ by S1P plays an important role in mediating the cardioprotective effects of FTY720 via down-stream PI3K signaling.

### Cardiac dysfunction following polymicrobial sepsis is attenuated by FTY720 treatment

**Figure 7:**
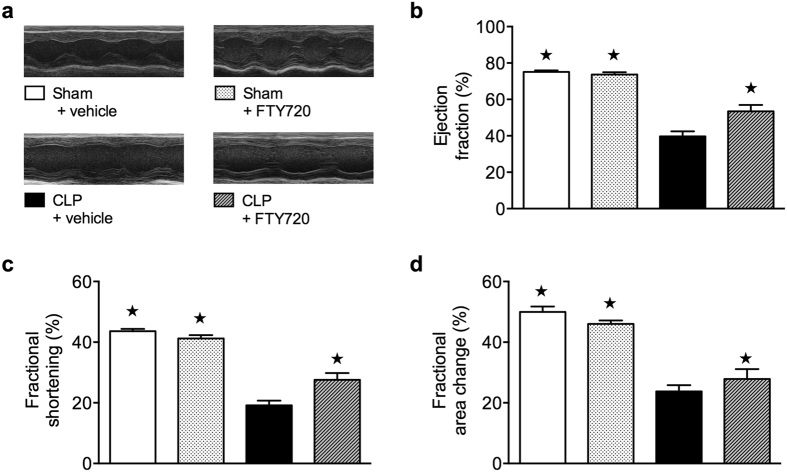
Effect of cecal ligation and puncture and treatment with FTY720 on cardiac function in C57BL/6J wild type mice. (**a**) Representative M-mode echocardiograms and legend of all groups studied. (**b**) Percentage ejection fraction, (**c**) fractional shortening (**d**), fractional area change were assessed via echocardiography 24 h subsequent to CLP or sham-operation in 8-month-old male C57BL/6J wild type mice. The following groups were studied: sham + vehicle (n = 8); sham + FTY720 (n = 3), CLP + vehicle (n = 8), CLP + FTY720 (n = 10). Data are expressed as means ± SEM for n number of observations. *P < 0.05 vs. CLP + vehicle (Man Whitney test, two-tailed).

Having shown that FTY720 prevents the impairment in cardiac function caused by LPS/PepG, we wished to confirm these findings in a model of polymicrobial sepsis (CLP with fluid resuscitation and antibiotics) mimicking the clinical syndrome (Fig. 7). We obtained no significant differences in percentage EF, FS and FAC in sham animals treated with FTY720 or vehicle. When compared to sham animals, mice that underwent CLP demonstrated a significant reduction in percentage EF (*P* = 0.0002), FS (*P* = 0.0002) and FAC (*P* = 0.0002), indicating impaired systolic contractility (Fig. 7). Delayed intravenous administration of FTY720 (0.1 mg/kg) 1 h after CLP significantly attenuated this impaired systolic contractility, indicated by a significant increase of percentage EF (*P* = 0.0117), FS (*P* = 0.0205) and FAC (0.0031) (Fig. 7).

### Effect of sepsis and treatment with FTY720 on Akt, eNOS and ERK1/2 phosphorylation in mouse heart tissue

**Figure 8:**
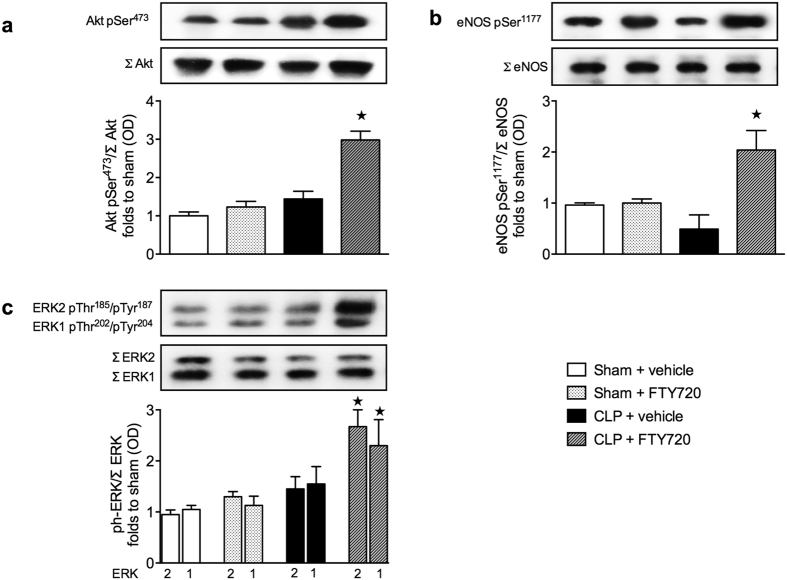
Effect of cecal ligation and puncture (CLP) and treatment with FTY720 on Akt, eNOS and ERK1/2 phosphorylation in murine heart tissue. At 1 h after CLP or sham operation, mice were treated with either FTY720 (0.1 mg/kg) or vehicle. Signaling events in heart tissue were assessed at 24 h. Each immunoblot is from a single experiment and is representative of three separate experiments. All values were corrected for the corresponding β-actin band. Densitometric analysis of the bands is expressed as relative optical density (OD) of (**a**) phosphorylated Akt (pSer^473^) corrected for the corresponding total Akt content (Σ Akt) and normalized using the related sham band; (**b**) phosphorylated eNOS (pSer^1177^) corrected for the corresponding total eNOS content (Σ eNOS) and normalized using the related sham band and; (**c**) phosphorylated ERK1 (pThr^202^/pTyr^204^) and ERK2 (pThr^185^/pTyr^187^) corrected for the corresponding total ERK1 or ERK2 content (ΣERK1 or Σ ERK2) and normalized using the related sham band. Data are expressed as mean ± SEM for *n* number of observations. *P < 0.05 vs. CLP + vehicle (Kruskall-Wallis test with Dunn´s multiple comparisons test).

Having shown that FTY720 activates Akt, eNOS and ERK1/2 in our model of MOD caused by LPS/PepG, we aimed to validate these findings in CLP-induced polymicrobial sepsis. When compared with heart tissues from sham mice treated with vehicle, heart tissues from sham mice treated with FTY720 and those from mice that underwent CLP and treated with vehicle demonstrated no significant alterations in the degree of phosphorylation of Akt on Ser^473^ (Fig. 8), eNOS on Ser^1177^ (Fig. 8) and ERK1/2 on Thr^202^/Tyr^204^ and Thr^185^/Tyr^187^ (Fig. 8), respectively. However, treatment of CLP challenged mice with FTY720 resulted in a significantly increased phosphorylation of Akt on Ser^473^ (*P* = 0.0232) (Fig. 8), eNOS on Ser^1177^ (*P* = 0.0092) (Fig. S3b) and ERK1/2 on Thr^202^/Tyr^204^ and Thr^185^/Tyr^187^ (*P* = 0.0100) (Fig. 8).

## Discussion

This study reports that serum S1P levels are significantly reduced in patients with sepsis and in animals challenged with LPS/PepG (reverse translation), which might contribute to septic cardiomyopathy. The lowest levels of S1P were found in patients that died in the acute phase of sepsis, while 28-day survivors appeared to have higher levels of serum S1P. Thus, our findings confirm and extend recently published data by Winkler *et al.*^17^. These authors investigated serum S1P levels in a larger cohort of patients with sepsis and found that serum S1P was decreased in sepsis and S1P levels negatively correlate with disease severity assessed by sofa score and 28-day mortality, however, their clinical study did not address the role of S1P in the pathophysiology of sepsis.

In mice, low levels of serum S1P were associated with a significant impairment in cardiac systolic contractility, and both pharmacological (FTY720) and genetic approaches (sphingosine kinase 2 deficiency) that increase serum S1P levels attenuated the cardiac dysfunction caused by LPS/PepG. These findings support our hypothesis that approaches, which enhance S1P serum levels, may reduce cardiac dysfunction and improve outcome in patients/animals with sepsis.

What, then, is the mechanism(s) by which high serum levels of S1P preserve cardiac function in sepsis? The S1P receptors S1P_1_, S1P_2_ and S1P_3_ activate the Akt survival pathway^30^ (Fig. 6a). When phosphorylated by it’s upstream regulator PI3K, Akt modulates inflammation, cell survival and growth^36^. Most notably, activation (resulting in phosphorylation of Ser^473^) of Akt attenuates the cardiac dysfunction caused by sepsis in mice^20^,^21^,^31^. Here, we show that the cardioprotective effects of FTY720 in sepsis are associated with increases in a) the serum levels of S1P and b) the phosphorylation of Ser^473^ on Akt resulting in the activation of the Akt survival pathway in the heart.

There is very good evidence that the activation of Akt results in the phosphorylation of eNOS (on Ser^1177^) and, hence, activation of eNOS^37^. Indeed, activation of S1P_1_, S1P_2_ and S1P_3_ activate the Akt/eNOS pathway. Activation of eNOS inhibits neutrophil adhesion, maintains microvascular patency^38^ and reduces the cardiac dysfunction in sepsis^20^,^21^,^31^. We have discovered that prevention of the cardiac dysfunction in sepsis afforded by FTY720 is associated with an increase in phosphorylation (on Ser^1177^) of eNOS. Thus, activation by FTY720/S1P of the Akt/eNOS survival pathway may contribute to the beneficial effects of FTY720/S1P in mice with sepsis. Activation of the S1P receptors S1P_1_, S1P_2_ and S1P_3_ also activates the ERK1/2 pathway^30^ (Fig. 6a). Activation of ERK1/2 promotes cell survival and proliferation^30^. Here we demonstrate that the cardioprotective effects afforded by FTY720 are associated with an increase in the phosphorylation (on Thr^202^/Tyr^204^ and Thr^185^/Tyr^187^) of ERK1/2 resulting in the activation of these kinases.

One could argue that the cardioprotection afforded by FTY720 is independent of a rise in serum S1P levels. To address this important question we have used a molecular approach to maintain elevated S1P levels in sepsis: Mice with a functional deletion of sphingosine kinase 2 have been reported to have higher endogenous S1P serum levels^27^,^28^,^29^. We show here that SPHK-2^−/−^ mice in comparison to wild type mice have a) elevated S1P serum levels b) exhibit no fall in S1P serum levels when challenged with LPS/PepG, and c) have less impairment in cardiac function when challenged with LPS/PepG. These findings further support the view that higher serum levels of S1P are associated with preservation in cardiac function in sepsis.

The activation of Akt/eNOS by the S1P receptors S1P_1_, S1P_2_ and S1P_3_ is dependent on the prior activation of PI3K (Fig. 6a). We report here that inhibition of PI3K (with LY294002) prevents the cardioprotective effects of FTY720 in sepsis (Fig. 6b). Thus, activation of PI3K (presumably secondary to the activation of the S1P receptor(s) by S1P) plays an essential role in the cardioprotective effects of FTY720/S1P.

We designed further experiments to gain a better understanding of the specific S1P receptor(s) that mediate the cardioprotective effects of FTY720. SEW2871 is a selective and specific agonist of S1P_1_^35^. SEW2871 did not significantly attenuate the cardiac dysfunction caused by LPS/PepG and, hence, did not mimic the effects of FTY720 in sepsis (Fig. 6b). Thus, it is less likely that the observed cardioprotective effects of FTY720 are secondary to the activation by S1P (or FTY720 itself) of the S1P_1_. In contrast, the S1P_2_ antagonist JTE 013^34^ abolished the cardioprotective effects of FTY720 indicating that these effects are (at least in part) secondary to the activation of S1P_2_ by S1P/FTY720 (Fig. 6b). It is possible that the activation of S1P_3_ also contributes to the cardioprotective effects of FTY720, as a) this receptor results in the activation of PI3K, and b) prevention of the activation of PI3K with LY294002 abolished the cardioprotective effects of FTY720. Thus, we provide evidence to suggest that the preserved cardiac function afforded by FTY720 in sepsis is at least in part secondary to the activation (by S1P) of S1P_2_ (and possibly S1P_3_). Interestingly, S1P activates Akt in cardiomyocytes and reduces the injury caused by myocardial ischemia-reperfusion, and both effects are lost in S1P_2_ and S1P_3_ double knock out mice^39^. Mechanistically, S1P_2_ and S1P_3_ are coupled to Ras homolog gene family member A (RhoA) activation^40^,^41^. RhoA activation protects cardiomyocytes against ischemia/reperfusion injury^42^,^43^. In addition, cardiomyocyte apoptosis is reduced *in vitro* by activation of the RhoA/Rho-associated protein kinase/Focal adhesion kinase/PI3K/Akt signaling pathway^44^. Furthermore, S1P can also mediate cardiomyocyte survival via activation of the RhoA signaling pathway involving RhoA, phospholipase C-ε and protein kinase D1^42^. Importantly, it should be noted that FTY720 lacks affinity for S1P_2_^45^, again supporting our view that the cardioprotective effects of FTY720 reported here are secondary to an increase in serum S1P levels after FTY720 treatment.

FTY720 is currently being used in the therapy of patients with multiple sclerosis^46^. Thus, it is, in principle, possible to evaluate the effects of FTY720 in patients with sepsis. Although systemic administration of TLR/NOD-ligands, such as LPS or PepG, can produce many of the features of sepsis including cardiac dysfunction^47^, CLP-sepsis is the model of choice when testing the efficacy of new therapeutics in sepsis. We show here that the delayed administration (after onset of CLP) of FTY720 in a clinically relevant, murine model of sepsis (CLP) with fluid resuscitation and antibiotic therapy attenuates the cardiac dysfunction caused by sepsis (Fig. 7). Interestingly, preservation of cardiac function in sepsis by FTY720 was associated with activation of the Akt/eNOS and ERK1/2 pathways (Fig. 8). These findings support the view that FTY720 attenuated the cardiac dysfunction in CLP-sepsis also in an Akt/eNOS and ERK1/2-dependent manner. FTY720 or S1P reduces the microvascular permeability in lung and kidney in mice challenged with LPS^16^ and FTY720 reduces plasma extravasation in rats with sepsis^48^. With this in mind, we have also investigated the effects of FTY720 on the renal dysfunction and liver injury associated with LPS/PepG. Although less pronounced, we observed a trend towards protection by FTY720, which failed to reach significance (see Supplementary Table S2). Thus, it seems likely that the acute beneficial effect of S1P elevation is restricted to the cardiovascular system.

What, then, is known about the role of endogenous S1P in patients with sepsis? Under normal conditions, the main portion of blood S1P is bound to HDL (60%) and serum albumin (35%) and only a minor amount is available as free S1P^49^. In sepsis, HDL levels are reduced^50^ and the application of HDL reduced mortality in various animal models of sepsis and multiple organ dysfunction^51^,^52^,^53^,^54^. The mechanism(s) underlying the protective effect of HDL are not completely understood, but may be due to direct binding and trapping of endotoxins by HDL^55^,^56^, the down-regulation of pro-inflammatory adhesion molecules and of chemotactic factors^51^. Additionally, and especially in view of our data here, the protective effects of HDL may be mediated by HDL-associated S1P. Interestingly, the many of the beneficial effects of HDL including the cardioprotective effects may be mediated by S1P (reviewed in^57^). It was recently discovered that apoM is the direct carrier of S1P in HDL^58^. Remarkably, apoM also decreases in sepsis^59^ supporting the view that the apoM-S1P-HDL may play a role in sepsis. Whether the reduction of apoM transcription in the septic liver^59^ is causal to the reduced serum S1P in sepsis remains open.

In conclusion, we confirmed patients with severe sepsis have lower serum levels of S1P and report here for the first time that in mice pharmacological (FTY720) and genetic (SPHK2 deficiency) approaches to enhance S1P serum levels reduce the cardiac dysfunction caused by LPS/PepG. In a model of polymicrobial sepsis we provide proof-of-concept for a potential therapeutic application of strategies to increase S1P. Preservation of cardiac function in sepsis by FTY720 is at least in part secondary to the activation (by S1P) of the S1P_2_ (and possibly S1P_3_) resulting in the PI3K-dependent activation of the Akt/eNOS and ERK1/2 pathways, which are known to be cardioprotective in animal models of sepsis. We speculate that FTY720 may be useful to elevate levels of S1P in patients with sepsis, which, in turn, may improve outcome in these patients. As FTY720 is used in patients with multiple sclerosis, it is, in principle, possible to evaluate the effects of this drug in patients with sepsis, although the known immunosuppressive effects of FTY720 need to be considered.

## Additional Information

**How to cite this article**: Coldewey, S. M. *et al.* Elevation of serum sphingosine-1-phosphate attenuates impaired cardiac function in experimental sepsis. *Sci. Rep.* **6**, 27594; doi: 10.1038/srep27594 (2016).

## Supplementary Material

Supplementary Information
