# Supplementary material for: Elevation of serum sphingosine-1-phosphate attenuates impaired cardiac function in experimental sepsis
Source: Sci Rep. 2016 Jun 9;6:27594. doi: 10.1038/srep27594 (PMC4899780; doi:10.1038/srep27594)
Supplement: Supplementary Information [file srep27594-s1.docx]

**Supplementary Information**

**Elevation of serum sphingosine-1-phosphate attenuates**

**impaired cardiac function in experimental sepsis**

Sina M. Coldewey, Elisa Benetti, Massimo Collino, Josef Pfeilschifter, Christoph Sponholz, Michael Bauer, Andrea Huwiler, Christoph Thiemermann

**METHODS**

**Reagents and compounds**

Reagents and compounds were purchased from Sigma Aldrich (Poole, Dorset, UK), unless otherwise stated. Antibodies for immunoblot analysis were purchased from Santa Cruz Biotechnology (Heidelberg, Germany). FTY720 was purchased from Cayman Chemicals (Ann Arbor, Michigan). LY294002, JTE013 and SEW2871 were purchased from Tocris Bioscience (Bristol, UK).

**Human materials**

**Table S1: Characteristics of patients enrolled.** APACHE II: Acute Physiology and Chronic Health Evaluation II, SAPS II: Simplified Acute Physiology Score, SOFA: “Sequential Organ Failure Assessment Score.

|  | ***n*** | **Age (y)** | **Height (cm)** | **Weight**  **(kg)** | **APACHE**  **II** | **SAPS** | **SOFA**  **mean** | **SOFA max** | **Gender (%) m/f** |
| --- | --- | --- | --- | --- | --- | --- | --- | --- | --- |
| **Control** | 11 | 64.7 ± 2.6 | 174.3 ± 2.8 | 81.8 ± 3.6 | 11.4 ±  1.3 | - | - | - | 90.9/  9.1 |
| **Sepsis** | 19 | 59.6 ± 3.1 | 168.4± 2.1 | 80.2 ± 3.9 | 24.0 ±  1.4 | 56.3 ± 4.6 | 10.5 ± 0.9 | 14.1  ± 0.8 | 63.2/  36,8 |
| **28-d survivors** | 9 | 59.3 ± 3.9 | 167.8 ± 2.8 | 73.8 ± 6.2 | 24.8 ±  1.7 | 57.7 ± 4.3 | 11.9 ± 0.8 | 14.7  1.1 | 55/  45 |
| **28-d non-**  **survivors** | 10 | 59.9 ± 3.9 | 169.1 ± 3.2 | 86.0 ± 4.4 | 23.2 ±  2.3 | 54.8 ± 8.0 | 9.2 ± 1.4 | 13.6  ± 1.32 | 70/  30 |

**Generation of sphingosine kinase deficient mice (SPHK-2^-/-^) mice**

SPHK-2^-/-^ mice were generated by Genoway (Lyon, France). Briefly, a targeting vector was generated containing at the 5’-end a Diphteria Toxin A negative selection cassette, a 5’ homologous region consisting of 6.1kb homologous *SphK2* sequences including exons 1-3, a Neo-cassette flanked by two LoxP sites instead of exons 4-7, and a 3’ homologous region consisting of 1.8kb targeted *SphK2* sequences including exon 8. Gene targeted embryonic stem cells were isolated to produce chimeric male mice that passed the disrupted allele to their offsprings. Heterozygous matings produced viable mice homozygous for the mutation. Mice were backcrossed to C57BL/6 in an accelerated backcrossing procedure by Charles River.

**Quantification of organ injury/dysfunction in mice**

Mice were anesthetized with ketamine (100 mg/kg bw)/xylazine (10 mg/kg bw) before being sacrificed. Blood was collected (cardiac puncture) and centrifuged (9900 *g* for 5 min). Serum was analyzed for serum creatinine, urea, aspartate aminotransferase (AST) and alanine aminotransferase (ALT) by IDEXX laboratories (Buckinghamshire, UK). Organ and serum samples were harvested, snap frozen and stored at -80 ° C.

**RESULTS**

**Effect of LPS/PepG co-administration and treatment with FTY720 on markers of renal function and hepatocellular injury in mice**

We measured serum urea and creatinine as markers for renal function as well as serum ALT and AST as markers for hepatocellular injury to characterize our model of LPS/PepG induced multiple organ dysfunction (MOD) and to evaluate potential effects of FTY720 administration on renal and hepatocellular injury. There were no significant differences in serum urea, creatinine, ALT and AST in sham animals treated with FTY720 or vehicle **(Tab. S2)**. When compared to the sham animals, mice subjected to LPS/PepG demonstrated a significant increase of serum urea and creatinine, indicating acute kidney injury, and, a significant increase of ALT and AST, indicating hepatocellular injury **(Tab. S2)**. After delayed intravenous administration of FTY720 (0.1 mg/kg) 1 h post LPS/PepG challenge, we observed a less pronounced protection regarding renal (urea, creatinine) and hepatocellular failure (transaminases) than in the heart, which failed to reach significance **(Tab. S2)**.

**Effect of polymicrobial sepsis and treatment with FTY720 on markers of renal function and hepatocellular injury in mice**

We measured serum urea and creatinine as markers for renal function as well as ALT and AST as markers for hepatocellular injury to characterize our model of polymicrobial sepsis induced by CLP. Furthermore we aimed to evaluate in true sepsis potential beneficial or even harmful effects of FTY720, which is clinically used as an immunomodulator for treatment of multiple sclerosis, There were no significant differences in serum urea, creatinine, ALT and AST in sham animals treated with FTY720 or vehicle **(Tab. S3)**. When compared to the sham animals, mice subjected to CLP demonstrated a significant increase of serum urea and a 2,84 fold increase of creatinine, indicating acute kidney injury, and, a significant increase of ALT and AST, indicating hepatocellular injury **(Tab. S3)**. After delayed intravenous administration of FTY720 (0.1 mg/kg) 1 h post CLP challenge, we observed a less pronounced protection regarding renal (urea, creatinine) and hepatocellular failure (e.g. transaminases) than in the heart, which failed to reach significance **(Tab. S2)**. However, we clearly demonstrate that treatment with the immunomodulator FTY720 does not have harmful side effects on hepatic and renal function in sham mice and in septic mice.

**FIGURES AND TABLES**

| **C57BL/6**  **2-month-old** | ***n*** | **Urea mmol/l** | **Creatinine (μmol/l)** | **ALT**  **(U/l)** | **AST**  **(U/l)** |
| --- | --- | --- | --- | --- | --- |
| **Sham**  **+ vehicle** | 8 | 8.55 ± 0.61★ | 30.28 ± 1.34★ | 32.23 ± 3.11★ | 111.86 ± 9.14★ |
| **Sham**  **+ FTY720** | 3 | 10.37 ±  1.3 | 29.47 ± 2.67★ | 27.60 ± 1.39★ | 91.1± 33.27★ |
| **LPS/PepG**  **+ vehicle** | 16 | 33.25 ± 1.39 | 48.31 ± 2.20 | 82.92 ± 7.76 | 212.53 ± 15.56 |
| **LPS/PepG**  **+ FTY720** | 15 | 29.53 ± 3.02 | 43.77 ± 3.73 | 65.27 ± 6.10 | 153.91 ± 12.96 |

**Table S2: Effect of LPS/PepG administration and treatment with FTY720 on markers on markers of renal function and hepatocellular injury in mice.** Creatinine: serum creatinine, ALT: serum alanine aminotransferase, AST: serum aspartate aminotransferase. ★ *P* < 0.05 vs. LPS/PepG + vehicle (Kruskall-Wallis test with Dunn´s multiple comparisons test).

| **C57BL/6**  **8-month-old** | ***n*** | **Urea mmol/l** | **Creatinine (μmol/l)** | **ALT**  **(U/l)** | **AST**  **(U/l)** |
| --- | --- | --- | --- | --- | --- |
| **Sham**  **+ vehicle** | 8 | 7.09 ±  0.55 ★ | 29.41 ± 1.39 | 52.99 ±  3.33★ | 240.6 ± 43.85★ |
| **Sham**  **+ FTY720** | 3 | 6.87 ± 0.61★ | 26.17 ± 0.90 | 36.53 ±  1.22★ | 196.0 ± 87.65★ |
| **CLP**  **+ vehicle** | 8 | 29.30 ± 3.35 | 55.36 ± 8.84 | 230.6 ± 15.21 | 621.7 ± 44.99 |
| **CLP**  **+ FTY720** | 10 | 26.20 ± 4.04 | 43.53 ± 2.1 | 206.1 ± 22.24 | 566.4 ± 69.93 |

**Table S3: Effect of CLP and treatment with FTY720 on markers on markers of renal function and hepatocellular injury in mice.** Creatinine: serum creatinine, ALT: serum alanine aminotransferase, AST: serum aspartate aminotransferase. ★ *P* < 0.05 vs. CLP + vehicle (Kruskall-Wallis test with Dunn´s multiple comparisons test).
